# Genome-Wide Comparative Analysis of the Phospholipase D Gene Families among Allotetraploid Cotton and Its Diploid Progenitors

**DOI:** 10.1371/journal.pone.0156281

**Published:** 2016-05-23

**Authors:** Kai Tang, Chun-Juan Dong, Jin-Yuan Liu

**Affiliations:** 1 Laboratory of Plant Molecular Biology, Center for Plant Biology, School of Life Sciences, Tsinghua University, Beijing, China; 2 Institute of Vegetables and Flowers, Chinese Academy of Agricultural Sciences, Beijing, China; USDA-ARS-SRRC, UNITED STATES

## Abstract

In this study, 40 phospholipase D (PLD) genes were identified from allotetraploid cotton *Gossypium hirsutum*, and 20 PLD genes were examined in diploid cotton *Gossypium raimondii*. Combining with 19 previously identified *Gossypium arboreum* PLD genes, a comparative analysis was performed among the PLD gene families among allotetraploid and two diploid cottons. Based on the orthologous relationships, we found that almost each *G*. *hirsutum PLD* had a corresponding homolog in the *G*. *arboreum* and *G*. *raimondii* genomes, except for *GhPLDβ3A*, whose homolog *GaPLDβ3* may have been lost during the evolution of *G*. *arboreum* after the interspecific hybridization. Phylogenetic analysis showed that all of the cotton PLDs were unevenly classified into six numbered subgroups: α, β/γ, δ, ε, ζ and φ. An N-terminal C2 domain was found in the α, β/γ, δ and ε subgroups, while phox homology (PX) and pleckstrin homology (PH) domains were identified in the ζ subgroup. The subgroup φ possessed a single peptide instead of a functional domain. In each phylogenetic subgroup, the PLDs showed high conservation in gene structure and amino acid sequences in functional domains. The expansion of GhPLD and GrPLD gene families were mainly attributed to segmental duplication and partly attributed to tandem duplication. Furthermore, purifying selection played a critical role in the evolution of PLD genes in cotton. Quantitative RT-PCR documented that allotetraploid cotton PLD genes were broadly expressed and each had a unique spatial and developmental expression pattern, indicating their functional diversification in cotton growth and development. Further analysis of *cis*-regulatory elements elucidated transcriptional regulations and potential functions. Our comparative analysis provided valuable information for understanding the putative functions of the PLD genes in cotton fiber.

## Introduction

Upland cotton *Gossypium hirsutum* is the world’s most valuable fiber crop [1, 2]. It has been widely grown in over 80 countries and accounts for more than 90% of commercial cotton production worldwide [3]. *G*. *hirsutum* is also studied as a model polyploid plant. It was a classic natural allotetraploid (AADD, 2n = 4x = 52) that arose from interspecific hybridization approximately 1 to 2 million years ago (mya) between the A genome diploid species *Gossypium arboreum* (AA, 2n = 2x = 26) and the D genome diploid species *Gossypium raimondii* (DD, 2n = 2x = 26) [2, 4]. In general, allopolyploid plants grow more vigorously and adaptively than their parents, and thereby allotetraploid cotton produces a higher yield and superior quality of fibers than their diploid progenitors under similar conditions [5]. Cotton fiber (commonly known as cotton lint) is the most important natural and renewable material for the textile industry and profoundly affects the world economy and human daily life [6].

Phospholipase D (PLD) is a major type of phospholipase in plants and catalyzes the hydrolysis of phospholipids at the terminal phosphodiester bond to produce a free head group and phosphatidic acid (PA) [7]. Two highly conserved HxKxxxxD (HKD) domains are the common feature of PLD proteins. The functional domains near the N-terminus are diverse. Some PLDs contained a Ca^2+^-dependent phospholipid-binding C2 domain, while others contained the phox homology (PX) and pleckstrin homology (PH) domains. Up until now, the PLD gene families have been identified in various plants [8–11]. In *Arabidopsis*, for example, 12 *AtPLD* genes were identified, encoding three PLDαs, two PLDβs, three PLDγs, one PLDδ, one PLDε and two PLDζs [8]. In the rice genome, 17 PLD genes were identified, encoding 14 C2-PLDs (α-, β-, γ-, δ- and ε- types PLDs), two PX/PH-PLDs (ζ-type PLDs), and one single peptide-PLD (SP-PLD, φ-type PLD), which possesses a signal peptide near the N-terminus instead of C2 or PX/PH domains [9]. Different PLDs are found to have different reaction requirements, lipid selectivity and subcellular location [12]. Increasing studies indicate that PLD and its product PA have been implicated in multiple plant growth and developmental processes, such as seed germination [13], seedling growth [14], pollen tube elongation [15], root hair growth [16], hypocotyl elongation[17] and leaf senescence [18]. PLD and PA are also found to play pivotal roles in signaling plant responses to various abiotic and biotic stresses, such as drought [19], salt [20], cold [21], light [14], wounding [22] and pathogen [23].

On the basis of comparative proteomic analysis in allotetraploid cotton, PLD might be a key enzyme participating in the regulation of secondary cell wall synthesis in cotton fibers [24]. Meanwhile, its product PA obviously accumulated at the initial stage of secondary cell wall thickening [25]. It was reported that PLD is responsible for a key event in signal transduction for the release of reactive oxygen species (ROS), particularly H_2_O_2_ via NADPH-oxidase [26]. It was also shown that a ROS burst occurs during the transition from elongation to secondary cell wall synthesis in developing fibers [27]. H_2_O_2_ functions as a developmental signal in the differentiation of secondary cell walls in fiber [28]. Thus, PLD might be directly involved in H_2_O_2_ accumulation at the early stage of secondary cell wall synthesis and may play an important role in the regulation of fiber development. In addition, PLD was also found to take part in the cold stress response in cotton fibers [21].

In our previous study, 19 *GaPLDs* were identified in the diploid cotton *G*. *arboreum*, and their expression profiles provided us some clues on functional diversity [29]. However, we still only have a limited understanding of expansion pattern, molecular evolution and functional diversification of this gene family in widely cultivated allotetraploid cotton. Recently, the *G*. *hirsutum* genome has been sequenced, opening a new chapter of cotton genomic studies [30, 31]. In this study, the PLD gene families were identified in *G*. *hirsutum* (*GhPLD*s) and *G*. *raimondii* (*GrPLD*s). Then, all of these *PLDs* were compared with the *GaPLD*s to evaluate the phylogenetic relationship, sequence characteristics, functional divergence and selective pressure analyses. In addition, the expression profiles of *GhPLD* genes in different allotetraploid cotton tissues were examined, and the *cis*-regulatory elements were also analyzed to account for the expression specificity and transcriptional regulation. This study will provide a better understanding of the expansion and diversification of the allotetraploid cotton PLD gene family and advance PLD functional research to enhance our ability to manipulate fiber and agronomic production of cotton.

## Materials and Methods

### Plant materials

Allotetraploid cotton (*G*. *hirsutum* cultivar ‘CRI 35’) was grown in the field under standard conditions at the Tsinghua University in China. When cotton plants were in full bloom (approximately 90 days after planting), we collected different cotton tissues, including roots, stems, leaves, petals, and stamens. Cotton fibers were harvested at 0, 5, 10, 15, 20 and 25 days post anthesis (dpa). All of these samples were immediately frozen in liquid nitrogen and then stored at -80°C until RNA extraction. Each sample was collected from 40 individual cotton plants.

### Database search for cotton PLD genes

*G*. *hirsutum* genome data were downloaded from the CottonGen database (http://www.cottongen.org) [30], and *G*. *raimondii* genome data were obtained from the Phytozome v9.1 database (http://www.phytozome.net/) [32]. To identify the *G*. *hirsutum* and *G*. *raimondii* PLD genes, the PLD protein sequences from *G*. *arboreum*, *Arabidopsis* and rice were used as queries in BLASTP search [33]. Moreover, the HMMER search was performed in the annotation database using the HKD domain (PF00614) as a keyword. All of the candidate PLD genes were conformed for two HKD domains by Pfam (http://pfam.sanger.ac.uk/search) and InterproScan databases (http://www.ebi.ac.uk/interpro/search/sequence-search).

### Sequence analysis methods

Multiple sequence alignment of PLD proteins was performed using Clustal W with standard settings [34]. The program MUSCLE was also used to perform multiple sequence alignments to confirm the ClustalW results [35].

Sequence identities of cotton PLDs at both the nucleotide and amino acid levels were calculated with the program DNASTAR Lasergene (http://www.dnastar.com/). The exon-intron structures of PLD genes were generated online by the Gene Structure Display Server (http://gsds.cbi.pku.edu.cn/).

The theoretical molecular weight (Mw) and the isoelectric point (p*I*) of the the deduced cotton PLD proteins were calculated by ExPASy (http://cn.expasy.org/tools). Subcellular localization was analyzed using the CELLO v2.5 server (http://cello.life.nctu.edu.tw/).

The *cis*-regulatory elements in the promoter sequences were analyzed using two publicly available databases: Database of Plant *Cis*-acting Regulatory DNA Elements (PLACE) (http://www.dna.affrc.go.jp/PLACE/) and the Plant CARE database (http://bioinformatics.psb.ugent.be/webtools/plantcare/html/).

### Phylogenetic tree construction

The program MEGA 6.0 was used to align the deduced amino acid sequences of cotton PLD genes to construct a phylogenetic tree [36]. A neighbor joining (NJ) consensus tree was constructed with the following parameters: P-distance, pairwise gap deletion, and bootstrap (1,000 replicates). Then, Maximum likelihood and Minimal Evolution methods of MEGA 6.0 were applied to validate the results from the NJ method. Meanwhile, Maximum Parsimony method of PHYLIP software [37] was also employed to create a new phylogenetic tree to validate the results from the NJ method. The tree topologies from different methods were very similar.

### Chromosomal location and gene duplication

The detailed physical positions of PLD genes were obtained from the CottonGen database and the Phytozome v9.1 database. Chromosomal localization and collinear relationships of genes were visualized using the program Circos [38].

Paralogous PLD genes were chosen from the results of sequence identity calculations and the phylogenetic tree. Gene duplication events were indicated by shared aligned sequence covering >70% of the longer gene and similarity of the aligned regions of >70%. A tandem duplication event has been defined as paralogous genes that are physically close to each other on the chromosomes [39]. A segmental duplication event has been defined as paralogous genes that result from large-scale events such as whole genome duplication or duplications of large chromosomal regions [40].

The *Ka* (nonsynonymous substitution rate) and *Ks* (synonymous substitution rate) values of the paralogous genes were estimated by the *KaKs*_Calculator [41]. Mean *Ks* could be used as the proxy for time and the conserved flanking protein-coding genes were also used to estimate dates of the segmental duplication events [42]. Based on the *λ* of synonymous substitution 1.5×10^−8^ substitutions/ synonymous site/year for cotton [43], the approximate age of duplicated events of the duplicate PLD gene pairs was estimated (Time = *Ks*/2*λ*). Moreover, the *Ka*/*Ks* ratio was used to show the selection pressure for the duplicate PLD genes. A *Ka*/*Ks* ratio >1, <1 or = 1 indicates positive, negative (purifying selection) and neutral evolution, respectively [44].

### RNA extraction and real-time quantitative RT-PCR

Total RNAs were extracted using the RNAprep pure plant kit according to the manufacturer’s instructions (TIANGEN, Beijing, China) from different cotton tissues. The first strand cDNAs were synthesized using the Takara Reverse Transcription System (TaKaRa, Shuzo, Otsu, Japan).

Quantitative real-time PCR (qRT-PCR) reactions were performed in Mini Opticon Real-Time PCR System (Bio-Rad, Hercules, California, USA) using the SYBR Green Master Mix Reagent (TaKaRa, Shuzo, Otsu, Japan) under standardized thermal cycling conditions according to the manufacturer’s protocol. Each PCR reaction (20 μL) contained 10 μL real-time PCR Mix, 0.5 μL of each gene-specific primer (S1 Table), and appropriately diluted cDNA. *GhUBQ7* (Accession number: DQ116441) was used as an internal reference gene. Each PCR reaction was repeated three times independently. The specificity of the reactions was verified by melting curve analysis, and products were further confirmed by agarose gel electrophoresis. The comparative 2^-ΔΔCT^ method was used to calculate the relative expression levels [45]. Heat maps were generated with the MultiExperiment Viewer (MeV) [46].

### RNA-sequencing data analysis

The high-throughput RNA-sequencing (RNA-seq) data of *G*. *hirsutum* for expression analysis in roots, stems, leaves, petals, stamens and fibers at 0, 5, 10, 20 and 25 dpa were downloaded from the National Center for Biotechnology Information Short Read Archive (http://www.ncbi.nlm.nih.gov/sra/) with the accession numbers SRX797899, SRX797900, SRX797901, SRX797903, SRX797904, SRX797909, SRX797917, SRX797918, SRX797919 and SRX797920, respectively [30]. The transcript abundance of each gene was calculated by the fragments per kilobase of exon model per million mapped reads (FPKM) with Cufflinks software (http://cufflnks.cbcb.umd.edu/). The log_2_(FPKM) values were utilized for generating the heat maps using the MeV software.

## Results

### PLD genes in *G*. *hirsutum* and *G*. *raimondii* genomes

The BLASTP and HMMER searches against *G*. *hirsutum* and *G*. *raimondii* genomes were performed to identify PLD genes. Then, the candidate PLD genes were confirmed through similarity searches against Pfam and InterproScan databases. Finally, a total of 40 *G*. *hirsutum PLDs* (*GhPLDs*) and 20 *G*. *raimondii PLDs* (*GrPLDs*) were identified (Table 1 and S2 Table). The properties of newly found cotton PLDs were analyzed by ExPASy and CELLO v2.5 servers. The ORF lengths of these genes ranged from 1,545 bp to 3,693 bp, which encoded polypeptides of 514 aa to 1,230 aa with predicted molecular weights ranging from 57.91 kD to 140.56 kD (Table 1). The theoretical p*Is* ranged from 5.37 to 9.13. Most of the PLDs were predicted to be localized in the cytoplasm (Table 1).

**Table 1:** The *PLD* genes in *G*. *hirsutum* and *G*. *raimondii* and properties of the deduced proteins.

Gene name	Gene ID	Chromosomal location^a^	ORF(bp)	Protein^b^			
				Size (aa)	MW (kDa)	p*I*	Subcellular localization^c^
*GhPLDα1A*	Gh_A10G0662	A10(-): 11048194–11066260	2,424	807	91.53	5.37	C
*GhPLDα1D*	Gh_D10G0730	D10(+): 8439309–8443570	2,424	807	91.53	5.48	C
*GhPLDα2A*	Gh_A06G1624	A06(-): 101165820–101169178	2,418	805	91.6	5.5	C
*GhPLDα2D*	Gh_D06G1993	D06(-): 61526855–61530236	2,418	805	91.55	5.55	C
*GhPLDα3A*	Gh_A07G0222	A07(+): 2700343–2703900	2,484	827	93.99	6.65	C
*GhPLDα3D*	Gh_D07G0279	D07(+): 2893589–2897211	2,484	827	94.07	6.76	C
*GhPLDα4A*	Gh_A05G0436	A05(-): 4839584–4842566	2,454	817	92.9	6.71	C
*GhPLDα4D*	Gh_D05G0560	D05(+): 4511885–4514866	2,454	817	93.08	6.62	C
*GhPLDβ1A*	Gh_A11G0758	A11(+): 7513190–7519138	3,375	1,124	125.68	7.7	N
*GhPLDβ1D*	Gh_D11G0886	D11(+): 7677837–7683822	3,375	1,124	125.53	7.2	N
*GhPLDβ2A*	Gh_A12G0942	A12(+): 59715352–59718967	2,355	784	88.99	8.16	C
*GhPLDβ2D*	Gh_D12G1032	D12(+): 36125870–36130292	3,168	1,055	119.57	8.28	N
*GhPLDβ3A*	Gh_A11G0761	A11(+): 7538255–7544383	3,390	1,129	125.42	6.7	N
*GhPLDβ3D*	Gh_D11G0888	D11(+):7700913–7707032	3,393	1,130	125.44	6.63	N
*GhPLDγ A*	Gh_A08G1600	A08(+): 94594467–94598013	2,556	851	95.54	8.19	C
*GhPLDγD*	Gh_D08G1911	D08(+): 57054075–57057621	2,556	851	95.9	7.6	C
*GhPLDδ1A*	Gh_A12G0540	A12(-): 13280804–13286284	2,550	914	96.46	6.69	C
*GhPLDδ1D*	Gh_D12G0556	D12(-): 10278949–10284459	2,550	914	96.37	6.69	C
*GhPLDδ2A*	Gh_A02G1674	A02(+):83053172–83057736	2,415	804	90.98	7.18	C
*GhPLDδ2D*	Gh_D03G0048	D03(+):312090–316688	2,571	856	96.97	6.63	C
*GhPLDδ3A*	Gh_A01G0880	A01(-):20697344–20701722	2,538	845	95.68	6.64	C
*GhPLDδ3D*	Gh_D01G0915	D01(-):15285684–15289947	2,538	845	95.78	6.72	C
*GhPLDδ4A*	Gh_A05G2168	A05(-):24844619–24854267	3,693	1,230	140.562	8.7	N
*GhPLDδ4D*	Gh_D05G2424	D05(-):24298286–24302113	2,595	864	98.669	8.44	N
*GhPLDδ5A*	Gh_A12G0243	A12(-):3722040–3726903	2,547	848	96.03	6.79	C
*GhPLDδ5D*	Gh_D12G0242	D12(-):3218723–3223600	2,550	849	96.12	7.03	C
*GhPLDε A*	Gh_A02G1124	A02(-):60940992–60943829	2,307	768	87.87	6.45	C
*GhPLDεD*	Gh_D03G0546	D03(-):10422279–10425127	2,307	768	87.72	6.75	C
*GhPLDζ1A*	Gh_A01G0213	A01(+):2083336–2091108	3,321	1,106	125.96	6.1	C
*GhPLDζ1D*	Gh_Sca005308G01	scaffold5308(+):923–8640	3,321	1,106	126.01	6.1	C
*GhPLDζ2A*	Gh_A10G1486	A10(+):81489677–81498158	3,345	1,114	126.71	6.33	N
*GhPLDζ2D*	Gh_D10G1729	D10(+):48605329–48613791	3,345	1,114	126.49	6.31	N
*GhPLDζ3A*	Gh_A02G0494	A02(+):7274495–7288655	2,919	972	109.9	6.28	N
*GhPLDζ3D*	Gh_D02G0554	D02(+):7493511–7502060	2,946	981	111.07	6.28	N
*GhPLDζ4A*	Gh_A11G2458	A11(+):83020505–83026075	3,327	1,108	126.32	5.57	C
*GhPLDζ4D*	Gh_D11G2775	D11(+):57479136–57489254	3,192	1,063	121.3	5.56	C
*GhPLDφ1A*	Gh_A07G0265	A07(-):3306792–3314618	1,545	514	57.91	7.23	E
*GhPLDφ1D*	Gh_D07G0321	D07(-):3400982–3403213	1,545	514	57.94	7.99	E
*GhPLDφ2A*	Gh_A06G0922	A06(-):37290132–37292468	1,380	459	52.17	8.98	PM
*GhPLDφ2D*	Gh_D06G1085	D06(-):24321096–24323423	1,623	540	61.55	9.13	PM
*GrPLDα1*	Gorai.011G083000.1	Chr11(+): 8380923–8385184	2,424	807	91.58	5.44	C
*GrPLDα2*	Gorai.010G225600.1	Chr10(-):59751136–59754500	2,418	805	91.51	5.63	C
*GrPLDα3*	Gorai.001G033300.1	Chr1(+):3069538–3073522	2,484	827	93.88	6.33	C
*GrPLDα4*	Gorai.009G057800.1	Chr9(+):4158704–4161686	2,454	817	93.14	6.55	C
*GrPLDβ1*	Gorai.007G094300.1	Chr7(+):6927130–6933259	3,393	1130	125.61	6.73	N
*GrPLDβ2*	Gorai.008G115700.1	Chr8(+):34857730–34862153	3,162	1053	119.23	7.59	N
*GrPLDβ3*	Gorai.007G094100.1	Chr7(+):6908403–6914399	3,375	1124	125.64	7.51	N
*GrPLDγ*	Gorai.004G206700.1	Chr4(+):53722628–53726174	2,556	851	95.93	7.6	C
*GrPLDδ1*	Gorai.008G061600.1	Chr8(-):9809029–9814536	2,550	849	96.25	6.69	C
*GrPLDδ2*	Gorai.003G005500.1	Chr3(-):302357–306946	2,565	854	96.8	6.55	C
*GrPLDδ3*	Gorai.002G120800.1	Chr2(-):17310003–17314270	2,562	853	96.72	6.59	C
*GrPLDδ4*	Gorai.009G268500.1	Chr9(-):22371067–22374881	2,523	840	95.74	8.6	PM
*GrPLDδ5*	Gorai.008G027300.1	Chr8(-):3218926–3223804	2,550	849	96.14	6.91	C
*GrPLDε*	Gorai.003G059700.1	Chr3(-):10351275–10354153	2,307	768	87.6	6.67	C
*GrPLDζ1*	Gorai.002G028800.1	Chr2(+):2142619–2150335	3,321	1106	125.91	6.21	C
*GrPLDζ2*	Gorai.011G193900.1	Chr11(+):46880104–46888580	3,300	1099	124.6	6.15	C
*GrPLDζ3*	Gorai.005G062100.1	Chr5(+):6558010–6566183	3,273	1090	124.04	6.18	N
*GrPLDζ4*	Gorai.007G302800.1	Chr7(+):51639889–51645452	3,237	1078	123.16	5.58	C
*GrPLDφ1*	Gorai.001G038100.1	Chr1(-):3551503–3553734	1,545	514	58	7.19	E
*GrPLDφ2*	Gorai.010G118800.1	Chr10(-):23679895–23682222	1,545	514	58.28	8.96	E

a. Chromosomal location: [ChrNo.(Orientation): start-end], ‘+’ and ‘-’ indicated the forward and reverse orientation, respectively.

b. The theoretical molecular weight (MW) and isoelectric point (p*I*) were calculated by ExPASy. Subcellular localization was analyzed using the CELLO v2.5 server. The Nucleic acid and deduced amino acid sequences of each *GhPLD* and *GrPLD* gene are listed in S2 Table.

c. C, cytoplasm; N, nucleus; PM, plasma membrane; E, extracellular space.

**Fig 1:**
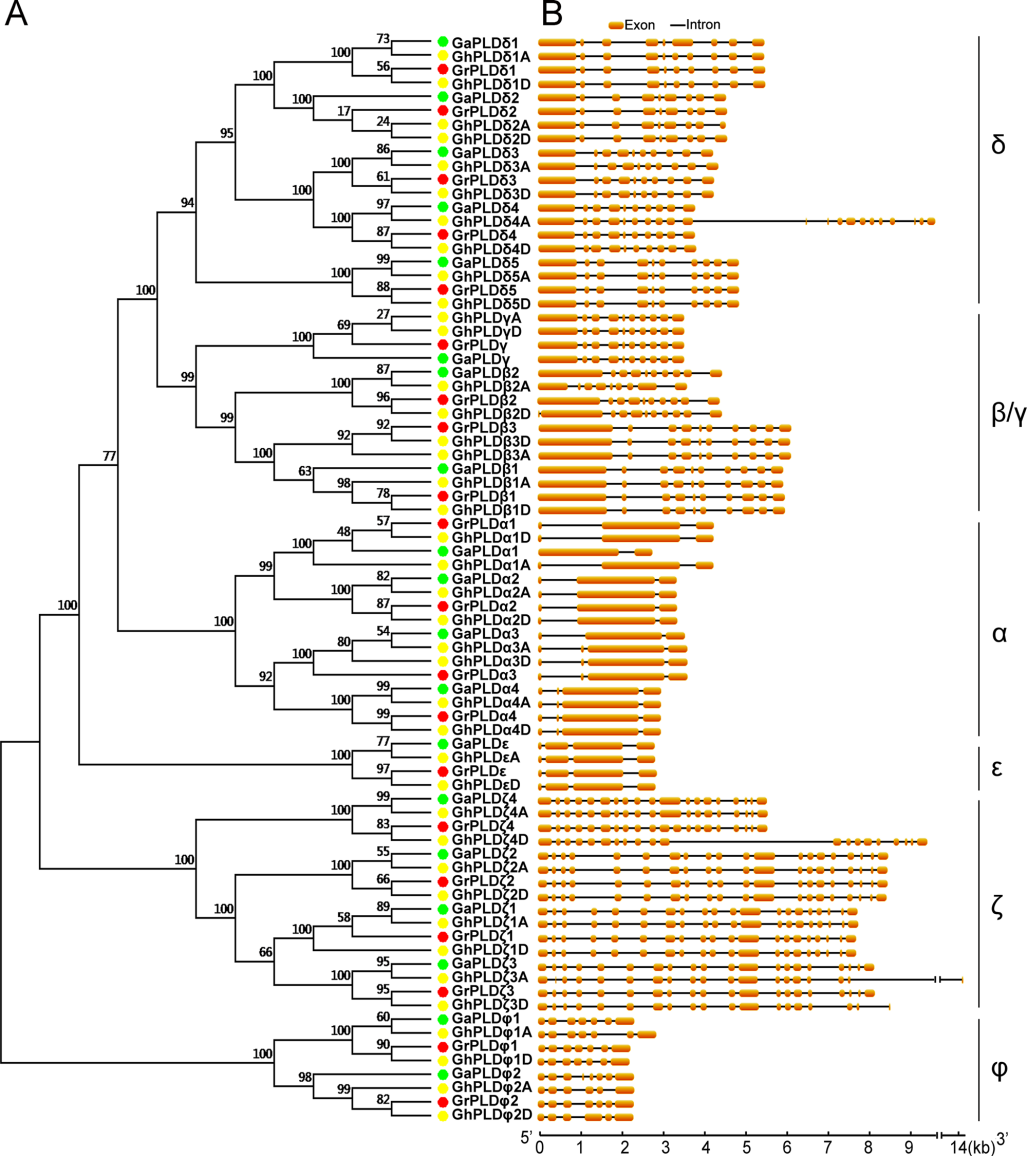
Phylogenetic relationship and gene structure of the cotton PLD genes. A. The phylogenetic tree was conducted using MEGA 6.0 software with the NJ method with bootstrapping analysis (1,000 replicates). The numbers beside the branches indicate the bootstrap values that support the adjacent nodes. Dots of different colors represented the different cotton species (Green, *G*. *arboreum*; red, *G*. *raimondii*; yellow, *G*. *hirsutum*). B. Schematic diagram for the exon-intron organization of cotton PLD genes. The orange boxes and black lines indicate the exons and introns, respectively.

The phylogenetic analysis allowed the classification of these cotton PLDs into six subgroups (α, β/γ, δ, ε, ζ and φ) (Fig 1A). According to the phylogenetic relationships with orthologs in *G*. *arboreum*, 20 GrPLDs were named as GrPLDα1-GrPLDα4, GrPLDβ1-GrPLDβ3, GrPLDγ, GrPLDδ1-GrPLDδ5, GrPLDε, GrPLDζ1- GrPLDζ4, GrPLDφ1 and GrPLDφ2 (Fig 1A and Table 1). Taking into account the gene location and orthologous relationship, we designated 40 GhPLDs as GhPLDα1A/α1D- GhPLDα4A/α4D, GhPLDβ1A/β1D-GhPLDβ3A/β3D, GhPLDγA/γD, GhPLDδ1A/δ1D-GhPLDδ5A/ δ5D, GhPLDεA/εD, GhPLDζ1A/ζ1D-GhPLDζ4A/ζ4D, GhPLDφ1A/φ1D and GhPLDφ2A/φ2D (Fig 1 and Table 1). Every GhPLD had its own orthology in both diploid relatives, with the exception of GhPLDβ3A. After attempting to amplify by specific primers and scanning the genome again, we still did not find a putative GaPLDβ3. Thus, we deduced that it might have been lost during the evolution of *G*. *arboreum* after interspecific hybridization.

Among these cotton PLD subgroups, the PLDδ constituted the largest, containing 20 members. The second largest subgroups (α and ζ) both consisted of 16 members. The subgroup β/γ was a unique bi-type subgroup which was comprised of 11 PLDβs and four PLDγs. In the subgroups φ and ε, eight and four members were identified, respectively (Fig 1A). Notablely, subgroups ζ and φ were far from the other four subgroups in evolutionary distance. Based on the functional domain prediction by searching the Pfam database, subgroups ζ and φ belonged to PX/PH-PLD and SP-PLD subfamilies, respectively, whereas the other subgroups fell into the subfamily C2-PLD. Overall, according to the phylogenetic relationships of cotton PLDs, it was speculated that these multiple subgroups might play specialized roles in the adaptive evolution of cotton.

### Sequence characteristics of cotton PLD genes

To understand phylogenetic relationships, we calculated the sequence identities of pairwise cotton PLDs at both the nucleotide and amino acid level and also compared the exon-intron structures of individual PLDs. The genes that belong to the same subgroup had high identity to each other, especially for the ones with an orthologous relationship (S1 Fig). For different subgroups, the nearer evolutionary distance they were in, the higher sequence identities they had, such as the PLDβ/γ and PLDδ subgroups (Fig 1A and S1 Fig). Significantly, four gene clusters (*PLDβ1s*-*PLDβ2s*- *PLDβ3s*, *PLDδ1s*-*PLDδ2s*, *PLDδ3s*-*PLDδ4s*, and *PLDφ1s*-*PLDφ2s*) with more than 90% identity existed in three subgroups, indicating that they might originate from gene duplication events (S1 Fig). The detailed illustration showed the distribution and position of introns within each of the cotton PLD genes (Fig 1B). A total of 680 introns were found in the cotton PLD genes, with an average intron number of 8.6 per gene and an average intron length of 258.9 bp (S3 Table). The members of the individual subgroups shared similar intron numbers, consistent with the phylogenetic classification of the cotton PLDs (Fig 1 and S3 Table). For example, both subgroups PLDβ/γ and PLDδ included members with approximately nine introns.

**Fig 2:**
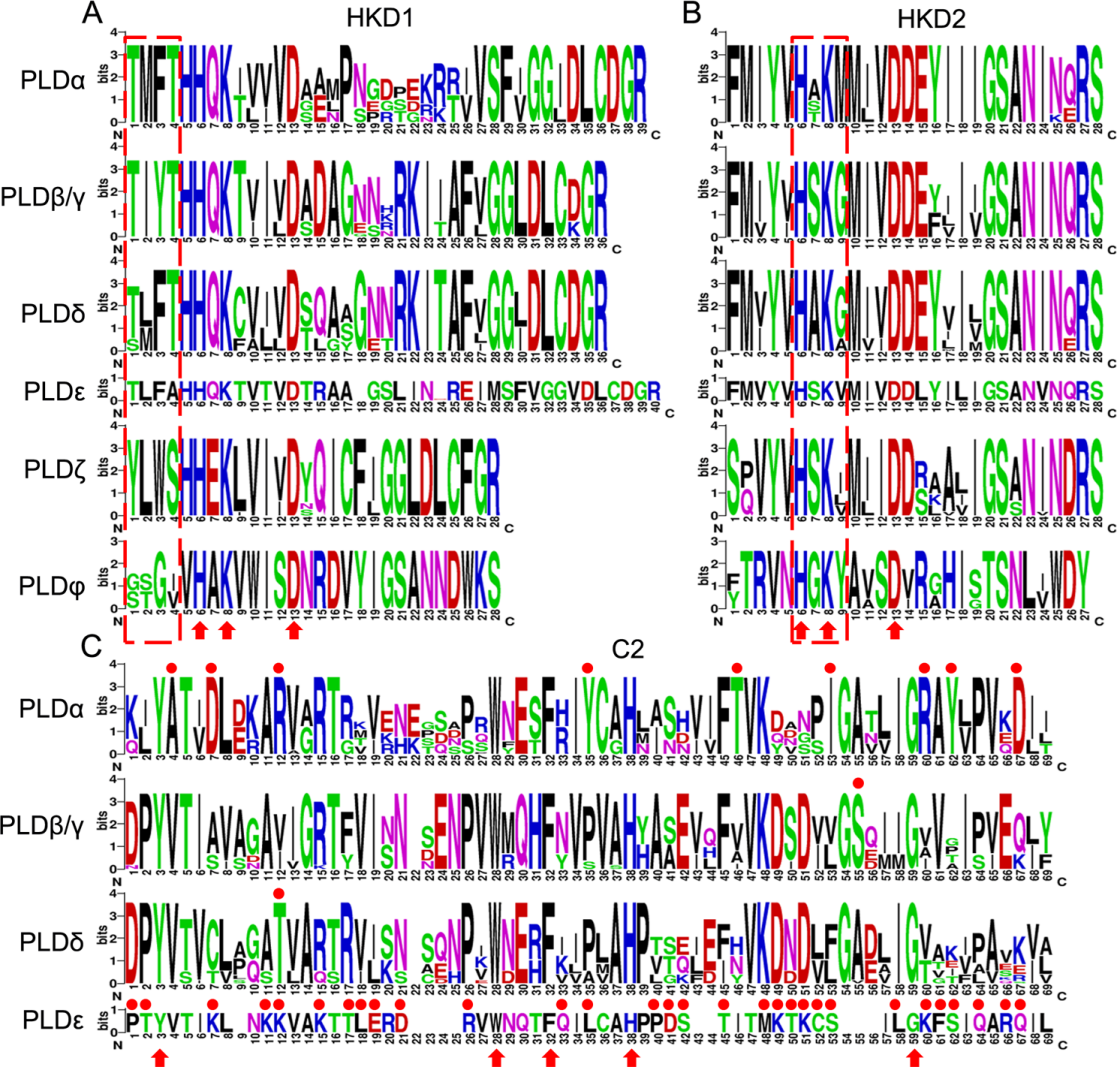
The conservation of amino acid residues in two HKDs and C2 domains were presented in different PLD subgroups. Numbers on the x-axis represent the sequence positions of each domain. The y-axis represents the information content measured in bits. A. Alignment of the HKD1 domains; B. Alignment of the HKD2 domains; C. Alignment of the C2 domains.

To reveal the typical domain characteristics of cotton PLD subgroups, comparative analyses for the conservation of amino acid residues in functional domains were performed on the basis of the alignments of these PLDs. The HKD1 domains were relatively more diverse than the HKD2 domains (Fig 2A and 2B). Both of the HKD1 and HKD2 domains contained three highly conserved amino acids (6H, 8K and 13D), which might have a key functional importance within these two domains (Fig 2A and 2B). The members of the individual subgroups possessed some marker amino acids in the HKD1 and HKD2 domains, for instance, “TMFT” and “H[A/ST]KM” in PLDαs, “TIYT” and “HSKG” in PLDβ/γs, “[T/S][L/M]FT” and “HAK[G/A]” in PLDδs, “TLFA” and “HSKV” in PLDεs, “YLWS” and “HSK[I/L/V]” in PLDζs, and “[G/S][S/T]G[IV]” and “HGKY” in PLDφs (Fig 2A and 2B). Similarly, the members of individual cotton PLD subfamilies also contained some specific amino acids, for example, “HHQK” in C2-PLDs, “HHEK” in PX/PH-PLDs, and “VHAK” in SP-PLDs (Fig 2A). For C2 domain sequences, five amino acids (3Y, 28W, 32F, 38H and 59G) are highly conserved, while some other amino acids were extremely specific for their own PLD subgroup, such as 4A, 7D, 12R, 35Y, 46T, 53I, 60R, 62Y and 67D for PLDα (Fig 2C).

### Gene duplication events in GhPLD and GrPLD gene families

**Fig 3:**
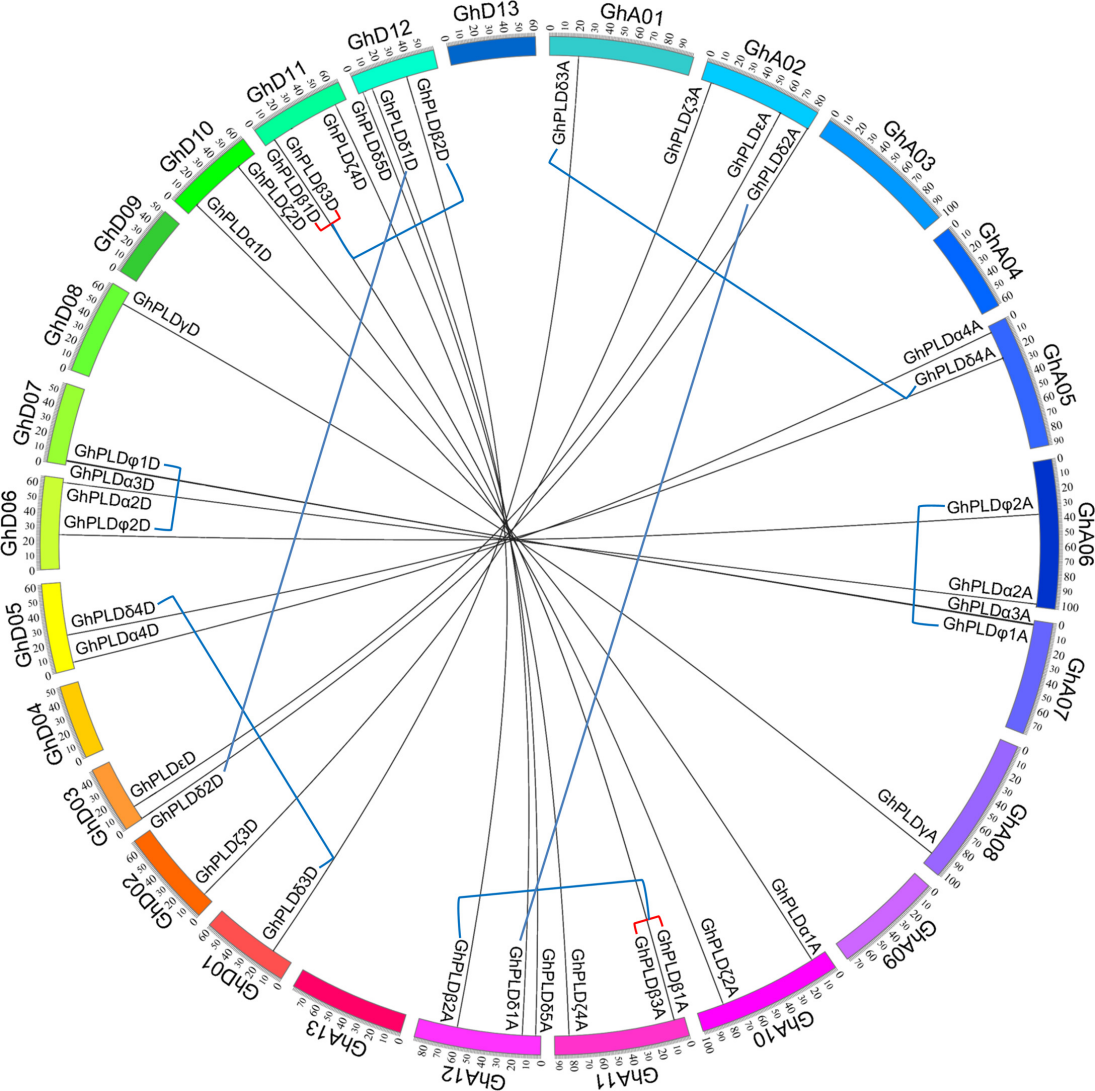
Syntenic relationship of the PLD genes in *G*. *hirsutum*. The syntenic relationships between *GhPLDxAs* and *GhPLDxDs* according to the CottonGen database are illustrated using the program Circos. Tandem and segmental duplicated PLD genes are connected by red and blue lines. The chromosomes of *G*. *hirsutum* are designated as GhA01—GhA13 and GhD01—GhD13.

After mapping all cotton PLD genes to their corresponding chromosomes, we found that they were unevenly distributed on the chromosomes of three cotton species (Fig 3 and S2 Fig). For the *GhPLD* gene family, 39 out of 40 PLD genes were assigned to 19 of the 26 *G*. *hirsutum* chromosomes (none were assigned to A03, A04, A09, A13, D04, D09 or D13), while one gene *GhPLDζ1D *was assigned to an as of yet unmapped scaffold (Fig 3). For the *GrPLD* gene family, all 20 PLD genes were assigned to 10 of the 13 *G*. *raimondii* chromosomes (none were assigned to Gr6, Gr12 or Gr13) (S2 Fig). We further assessed the contribution of gene duplication to the expansion of these two cotton PLD gene families. In *G*. *hirsutum*, eight segmental duplications (*GhPLDβ1A*/*β2A*, *GhPLDδ1A*/*δ2A*, *GhPLDδ3A*/*δ4A*, *GhPLDφ1A*/*φ2A*, *GhPLDβ1D*/*β2D*, *GhPLDδ1D*/*δ2D*, *GhPLDδ3D*/*δ4D* and *GhPLDφ1D*/*φ2D*) and two tandem duplications (*GhPLDβ1A*/*β3A* and *GhPLDβ1D*/*β3D*) occurred from 17.88 to 28.01 mya (Fig 3 and S4 Table). In *G*. *raimondii*, four segmental duplication (*GrPLDβ1*/*β2*, *GrPLDδ1*/*δ2*, *GrPLDδ3*/*δ4* and *GrPLDφ1*/*φ2*) and one tandem duplication (*GrPLDβ1*/*β3*) jointly took place during the time of 18.06~21.93 mya (Fig 3 and S4 Table). All identified gene duplication events in the three cotton PLD families happened before the split of two diploid cottons. Thus, we speculate that an unidentified tandem duplication event also happened previously in *G*. *arboreum*, followed by a specific gene loss event of *GaPLDβ3*.

To investigate which type of selection pressure had been involved in the divergence after gene duplication events, we calculated the *Ka*/*Ks* ratios (synonymous substitutions to non-synonymous substitutions) for the duplicated cotton PLD gene pairs on the basis of coding sequences. The resulting pairwise comparison data showed that all of the paralogous genes had *Ka*/*Ks* ratios of < 1, suggesting that cotton duplicated PLD genes had experienced strong purifying selection pressure. Strong functional constraints had a bearing on the evolution of these gene families, reflecting the essential roles of PLDs in cotton.

### Spatial expression profiles of GhPLD genes

To better reveal the potential functions of PLDs in allotetraploid cotton, the expression profiles of *GhPLDs* were investigated by both quantitative RT-PCR and RNA-seq data analysis. Because of the extremely high similarity between the mRNAs of the *GhPLDxA*-*GhPLDxD* gene pairs (such as *GhPLDα1A* and *GhPLDα1D*) and their nearly identical transcript sizes, we could not distinguish them using quantitative RT-PCR. Therefore, we regarded *GhPLDxA*-*GhPLDxD* as one combination named *GhPLDx* and investigated its expression level by quantitative RT-PCR, and then distinguished the portion of *GhPLDα1A* and that of *GhPLDα1D* by analyzing RNA-seq data, which included both gene expression levels and the information of gene location. Alternatively, the expression profiles of *GhPLD*s obtained from qRT-PCR and semi-quantitative PCR, were confirmed by RNA-seq data analysis.

**Fig 4:**
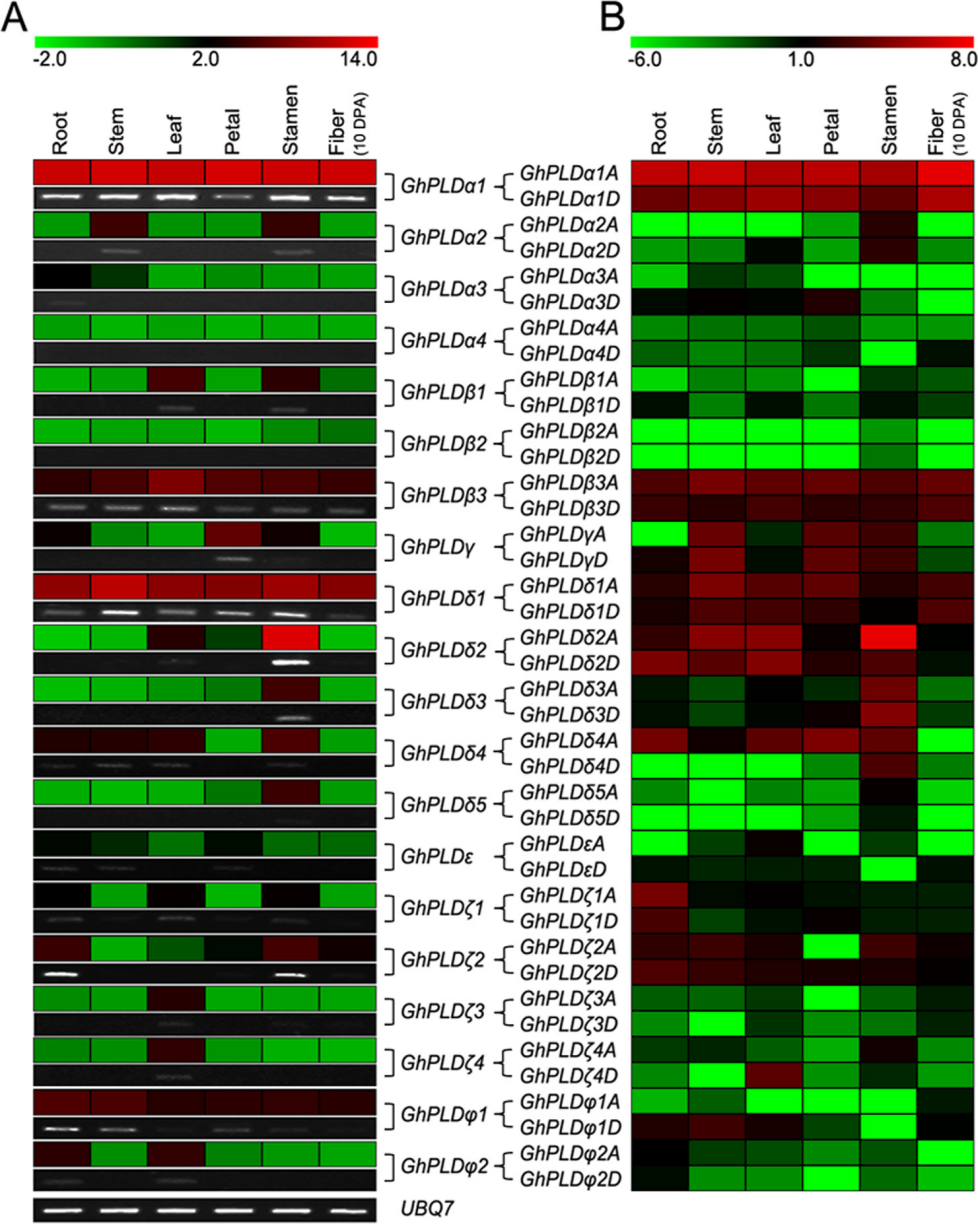
Expression patterns of *GhPLDs* in different cotton tissues. Expression patterns of *GhPLDs* in roots, stems, leaves, petals, stamens, and 10 DPA fibers. A. Real-time quantitative RT-PCR and semi-quantitative PCR results. B. RNA-seq data analysis results. The Illumina reads for expression analysis in cotton roots, stems, leaves, petals, stamens and 10 DPA fibers are retrieved from the NCBI SRA database. The color scale at the top of the left column heat map indicates the relative expression levels where light green indicates low and red indicates high. The color scale at the top of the right column heat map represents the FPKM values normalized log2 transformed counts where light green indicates low and red indicates high.

We found that *GhPLD* genes have rather broad expression patterns across a variety of cotton tissues under normal growth conditions including roots, stems, leaves, petals, stamens and fibers. The gene combination from the PLDα subgroup, *GhPLDα1*, had the most preferential expression levels in roots, stems, leaves and fibers and maintained high levels of expression in petals and stamens (Fig 4A). *GhPLDδ2*, with the highest expression abundance only in stamens, belongs to the largest subgroup (PLDδ). Another gene combination from this subgroup, *GhPLDδ1*, showed the second highest expression level in almost all cotton tissues, and *GhPLDβ3*, which belongs to the PLDβ/γ subgroup, expressed the third highest levels (Fig 4A). Similarly, *GhPLDζ2* and *GhPLDφ1*, which were from the *PLDζ* and *PLDφ* subgroups, respectively, were expressed at moderate levels, while the only gene combination of the remaining subgroup, *GhPLDε*, showed low expression values in all tissues (Fig 4A). Other low expression genes included *GhPLDα2*, *GhPLDβ1*, *GhPLDγ*, *GhPLDδ3*, *GhPLDδ4*, *GhPLDζ1*, *GhPLDζ3*, *GhPLDζ4* and *GhPLDφ2*. However, *GhPLDα3*, *GhPLDα4* and *GhPLDβ2* had only weak expression or even undetected expression (Fig 4A). Additionally, the duplicated *GhPLD* gene pairs displayed variant expression patterns. For instance, *GhPLDβ2* had almost undetected expression in all tissues, while *GhPLDβ1* maintained obvious expression levels in leaves and stamens. Examining when and where a gene is expressed in the plant tissues/organs can often lead to clues about gene function. Therefore, these diverse expression patterns of *GhPLDs* allude to functional diversification of this gene family in allotetraploid cotton.

To gain deeper insights into the expression patterns, we analyzed a RNA-seq dataset that encompassed results from six studied cotton tissues. In these surveyed gene combinations, we successfully distinguished the contribution of *GhPLDxAs* and that of *GhPLDxDs*. Most *GhPLDxAs* were more preferentially expressed than *GhPLDxDs* (Fig 4B). For instance, *GhPLDα1A* was expressed more preferentially than *GhPLDα1D* in all studied cotton tissues. However, the opposite correlation was observed for *GhPLDφ1D*, which maintained higher expression levels than *GhPLDφ1A*. Overall, the results of RNA-seq expression data were in close agreement with that of quantitative RT-PCR (Fig 4B).

### Expression patterns of GhPLD genes in developing fibers

**Fig 5:**
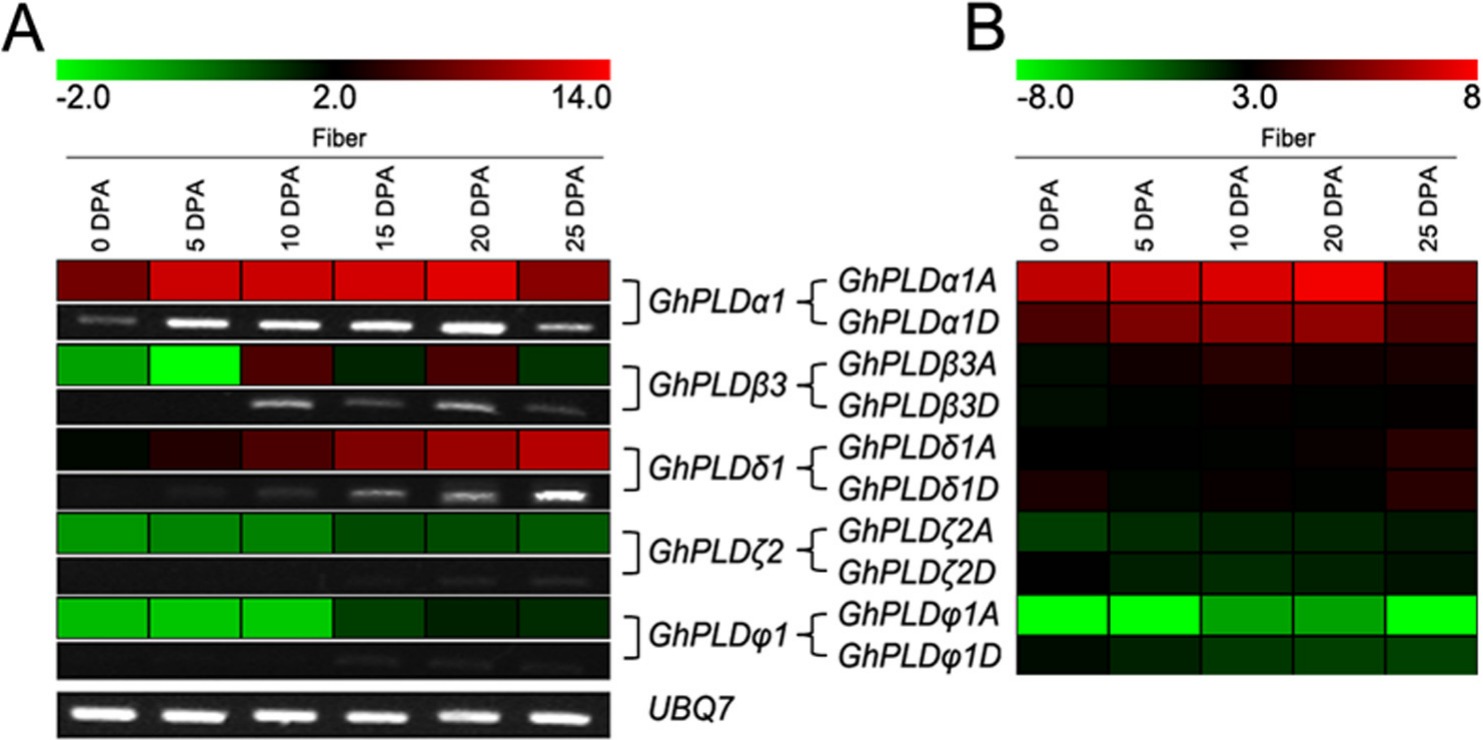
Expression patterns of *GhPLDs* in developing fibers. Expression patterns of *GhPLDs* were analyzed in cotton fibers at different developmental stages (0, 5, 10, 15, 20, and 25 DPA). A. Real-time quantitative RT-PCR and semi-quantitative PCR results. B. RNA-seq data analysis results. The Illumina reads for expression analysis in developing fibers (0, 5, 10, 20 and 25 dpa) are retrieved from the NCBI SRA database. The color scale at the top of the left column heat map indicates the relative expression levels where light green indicates low and red indicates high. The color scale at the top of the right column heat map represents the FPKM values normalized log2 transformed counts where light green indicates low and red indicates high.

It is worth noting that the *GhPLDs* that were expressed in fibers, which is the most valuable economic tissue in cotton, were *GhPLDα1A/D*, *GhPLDβ3A/D*, *GhPLDδ1A/D*, *GhPLDζ2A/D* and *GhPLDφ1A/D*. To survey the expression profiles of these fiber expressed PLD genes, quantitative RT-PCR and RNA-seq data analysis were performed at the representative stages of fiber development (0~25 DPA). Strikingly, among these selected genes, *GhPLDα1* had high expression in elongating fibers. Specifically, expression increased from 0 to 20 DPA, peaking at 20 DPA, and then decreased substantially (Fig 5A). Furthermore, *GhPLDα1A* made more of a contribution than *GhPLDα1D* did at every studied stages of fiber elongation (Fig 5B). These results implied that *GhPLDα1A* and *GhPLDα1D* might jointly play an important role in fiber development, most likely in the initiation stage of secondary cell wall thickening in fiber. In addition, *GhPLDδ1A* and *GhPLDδ1D* may also have an essential role in fiber development around the time of 25 DPA (Fig 5). However, *GhPLDβ3A*, *GhPLDβ3D*, *GhPLDζ2A*, *GhPLDζ2D*, *GhPLDφ1A* and *GhPLDφ1D* had relatively low or even undetectable expression levels in elongating fibers (Fig 5).

### *Cis*-regulatory elements in the promoters of GhPLD genes

**Fig 6:**
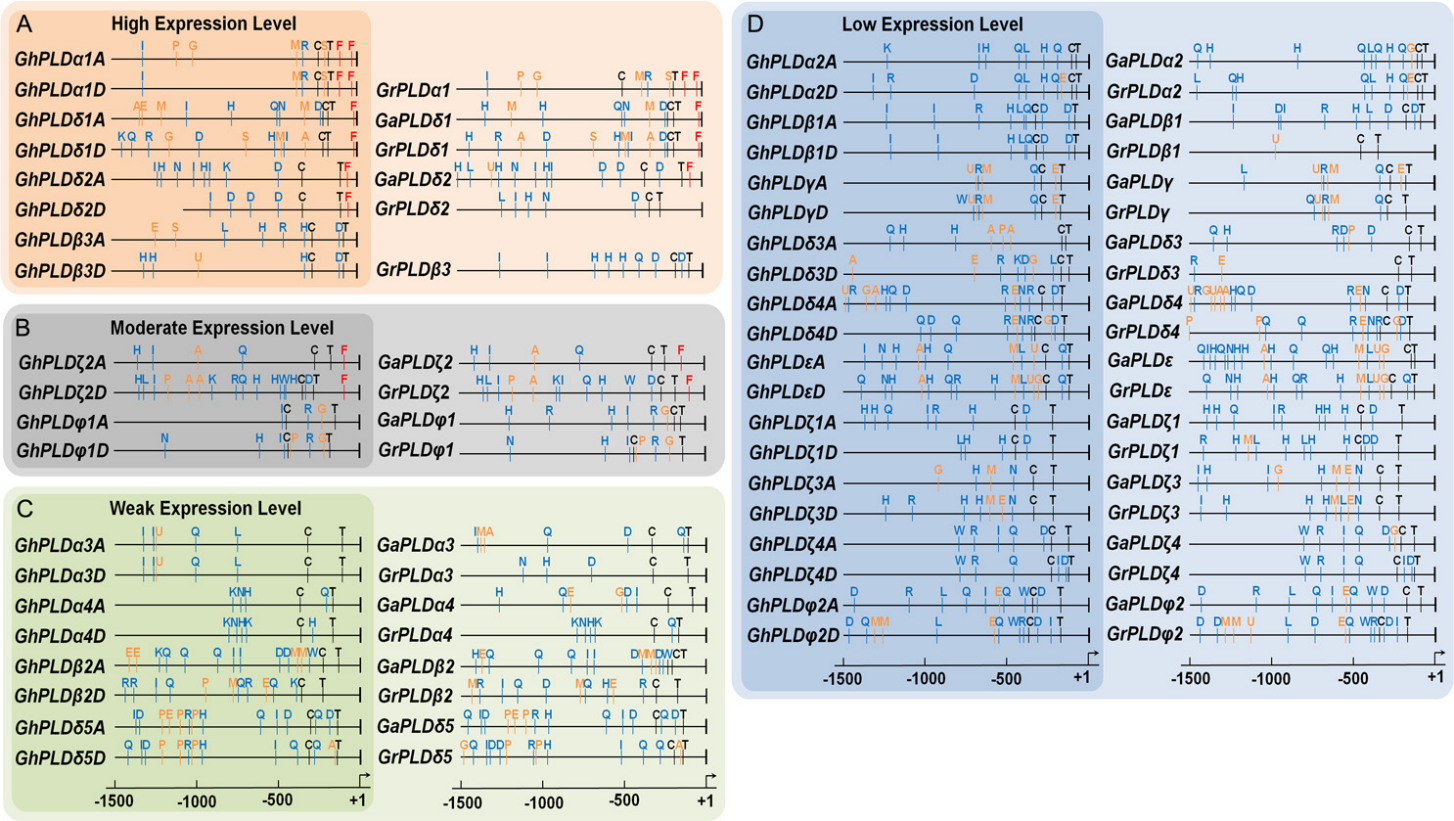
Putative regulatory *cis*-elements in the PLD gene promoters of cotton. The relative positions of elements are labeled with capital letters in the figure and are denoted in S6 Table. The hormone response *cis*-elements are in orange, the stress response *cis*-elements are in blue, and the high transcription *cis*-elements are in red.

To obtain more insights into the expression patterns and putative functions, the *cis*-regulatory elements were scanned in the promoter regions of *GhPLDs*. According to the expression levels, we divided 40 *GhPLDs* into four groups, including high, middle, low and weak expressed gene groups. Putative promoter regions in the 1500 bp sequences upstream of the translational start site were used to identify putative *cis*-regulatory elements. Moreover, the PLD genes of two diploid cottons were also sent to analyze the distributions and positions of the *cis*-regulatory elements. In addition to two core *cis*-elements (TATA box and CAAT box), one high transcription related element, six phytohormone response elements and nine stress response elements were found (S6 Table and Fig 6).

Most of the gene combinations (*GhPLDxA*-*GhPLDxD)* shared the similar *cis*-regulatory element organization, except for *GhPLDζ2A*-*GhPLDζ2D*, *GhPLDβ2A*-*GhPLDβ2D* and *GhPLDδ3A*- *GhPLDδ3D* (Fig 6). It was also shown that most of the identified *cis*-elements were found to be preserved in three studied cotton species (Fig 6). The conservation of *cis*-regulatory elements reflected the importance of meaningful transcriptional regulation of cotton PLDs. Remarkably, the element Py-rich stretch (F) existed in the 5`UTR regions of most of the highly expressed *GhPLDs*. The most preferentially expressed *GhPLDα1A* and *GhPLDα1D* had even two copies, suggesting that this regulatory element might direct the high-level expression (Fig 6A). Moreover, in all of the studied cotton tissues, *GhPLDα1A* and *GhPLDφ1D* expressed more preferentially than *GhPLDα1D* and *GhPLDφ1A*, respectively. For these two gene pairs, the differences in the promoters were the GA response elements P-box and GARE, which might be the key factors of expression level (Figs 4 and 6).

## Discussions

### Expansion of the allotetraploid cotton PLD gene family

In this study, the analyses of phylogeny, sequence characteristics and gene duplication were integrated to estimate an expansion pattern of allotetraploid cotton PLDs (S3 Fig). Our results have illustrated that the expansion was primarily due to a recent cotton specific large-scale genome duplication event, with tandem duplication and segmental duplication jointly taking place at some locations. These allotetraploid cotton PLDs were divided into six different subgroups with distinct sequence characteristics. Interestingly, the positions of introns in each subgroup were well conserved, indicating that the members of the same subgroup might have a common ancestor (Fig 1). PLDβ/γs and PLDδs had the closest evolutionary relationship and a similar distribution of exon-intron organization and could form a larger clade, implying that they might originate from the common ancestor I (Fig 1 and S3 Fig). PLDαs and PLDεs possessed 2 or 3 introns, suggesting that they originated from the common ancestor II (Fig 1 and S3 Fig). In addition, of these PLDs, PLDζs and PLDφs belonged to the PX/PH-PLD and SP-PLD subfamilies, respectively, and had dissimilar intron numbers, suggesting the convergent evolution via two independent evolutionary paths (Fig 1 and S3 Fig). Despite specific features of the functional domains near the N-terminus, the sequence identities of the C-terminus were relatively conserved, especially near the two characteristic HKD domains [47] (Fig 2). Thus, we reasonably speculated that all plant PLDs might have evolved from one original ancestor followed by some unknown changes that took place near the N-terminus, dividing the plant PLD gene family into three distinct PLD subfamilies: C2-PLD, PX/PH-PLD and SP-PLD (S3 Fig).

In the long history of evolution, selection pressure after gene duplication events shaped gene families, resulting in distinct evolutionary patterns among different gene families and even different subgroups in one gene family [48]. The members of the subgroup PLDφ were believed to be more conserved than those of the subgroups PLDβ/γ and PLDδ, as they had higher degrees of sequence identity and more similar exon-intron structures than PLDβ/γs and PLDδs (Fig 1 and S1 Fig). This was consistent with our analysis on the rate of molecular evolution, in which the mean values of *Ka*/*Ks* ratios in the subgroup PLDφ were smaller than those in the PLDβ/γ and PLDδ subgroups (S5 Table).

### Functional diversification of allotetraploid cotton PLD genes

Increasing evidence suggests the PLDs are involved in many plant processes including plant development, responses to biotic and abiotic stresses. *Cis*-regulatory elements in gene promoter regions have essential roles in determining tissue-specific and stress-responsive expression patterns of genes [49], which might help to elucidate transcriptional regulation and potential functions of *GhPLD*s. Phytohormones are important regulators of plant growth and development. Cotton fiber development is known to be regulated by gibberellic acid (GA) [50], jasmonic acid (JA) [51], abscisic acid (ABA) [52], ethylene [53] and auxin [54]. Among the 10 *GhPLD* genes expressed in elongating allotetraploid cotton fibers, 5, 4, 4, 2 and 1 genes were found to have *cis*-regulatory elements (P-box, GARE, TGACG-motif, ABRE, ERE and TGA-element) specific for GA, JA, ABA, ethylene and auxin responses in their respective promoters (S6 Table and Fig 6). Three preferentially expressed genes in fiber, including *GhPLDα1A*, *GhPLDδ1A* and *GhPLDδ1D*, all had more than three types of hormone response elements, strongly suggesting that the functions of these *GhPLD*s in elongating cotton fibers were synergistically regulated by different phytohormones. Other tissue-specific *GhPLDs*, such as *GhPLDγA*, *GhPLDγD*, *GhPLDδ4A* and *GhPLDδ4D*, possessed 2~4 types of hormone response elements, indicating that multiple phytohormones also regulated the transcription of *GhPLD*s in other cotton tissues.

Stress response elements related to diverse environmental stimuli were found in the promoter regions of allotetraploid cotton PLD genes (S6 Table and Fig 6). For the vast majority of *GhPLDs*, except for the preferentially expressed genes, the distribution of elements for stress responsiveness was more extensive, explaining why most of the *GhPLDs* exhibited the relatively low or weak expression levels under normal growth conditions (Figs 4 and 6). Adverse environmental stimulus, such as drought, defense, and heat shock, might trigger high levels of transcription of most low or weak expressed *GhPLDs*, as a result of that a number of the specific elements (MBS, TC-rich and HSE) were located in the promoters of these genes (Fig 6C and 6D). Therefore, we hypothesize that *GhPLDs* might also have important functions in the adaptation to adverse environmental stimuli. Overall, complex and diverse transcriptional regulation significantly broadened our view and understanding of the functional diversification of allotetraploid cotton PLDs.

To date, the biological and cellular functions of GhPLD genes remain largely unknown. The current investigation demonstrates some of GhPLD genes that might be involved in cotton development and stress response, and provides important clues for the selection of candidate genes, especially *GhPLDα1A/D* and *GhPLDδ1A/D* for the further studies.

## Conclusions

In conclusion, a total of 40 and 20 PLD genes were identified in *G*. *hirsutum* and *G*. *raimondii*, respectively. Our comparative analyses provided valuable insight into the understanding of phylogenetic relationships, sequence characteristics, molecular evolution of PLD genes in allotetraploid cotton and its two diploid progenitors. Moreover, we also characterized the broad spatial and fiber developmental expression profiles of allotetraploid cotton PLDs and expanded the view of transcriptional regulation of these genes. Unveiling the roles of PLD genes in cotton growth, development and stress adaptation processes may facilitate advances in crop variety development and utilization.

## Supporting Information

S1 Fig Sequence identity of the cotton PLD genes. The sequence identities of cotton PLDs at both the nucleotide and amino acid level are calculated with the program DNASTAR. The color scale at the top of the heat map indicates the levels of the sequence identities where light green indicates low and red indicates high. The data at the diagonal lines are equal to 100%.
(TIF)

S2 Fig Syntenic relationship of the PLD genes in *G*. *arboreum* and *G*. *raimondii*. The syntenic relationships between *GaPLDs* and *GrPLDs* according to the CottonGen and the Phytozome v9.1 database are illustrated using the program Circos. Tandem and segmental duplicated PLD genes are connected by red and blue lines. The chromosomes of *G*. *arboreum and G*. *raimondii* are designated as Ga1—Ga13 and Gr1—Gr13, respectively.
(TIF)

S3 Fig The expansion of the allotetraploid cotton PLD family. The left column represents the phylogenic relationships of allotetraploid cotton PLDs constructed using the NJ method. Letters in the circles indicate the identified gene duplication event (TD, tandem duplication; SD, segmental duplication). The middle column is the representative exon-intron organization of the PLD subgroup. PLDxA and PLDxD represent *G*. *hirsutum* PLDxA and *G*. *hirsutum* PLDxD genes, respectively. The right column indicates the putative common ancestors of allotetraploid cotton PLD subgroups.
(TIF)

S1 Table Primers used for qRT-PCR and semi-quantitative PCR. (XLSX)

S2 Table Nucleic acid, deduced amino acid, promoter and genomic sequences of *GhPLD* and *GrPLD* genes. (XLSX)

S3 Table Intron number and average length of cotton PLD genes in each subgroup. (XLSX)

S4 Table Duplicated *GhPLDs* and *GrPLDs* and the numbers of conserved protein-coding genes flanking them. Abbreviation: *Ks*-synonymous substitution rates; SD *Ks*-Standard deviation *Ks*; Mini *Ks*-Minimum *Ks*; Max *Ks*-Maximum *Ks*; mya-million years ago.
(XLSX)

S5 Table The *Ka*/*Ks* ratios for duplicate PLD genes in *G*. *arboreum*, *G*. *raimondii*, and *G*. *hirsutum*. (XLSX)

S6 Table Putative *cis*-element sequences in promoter regions of cotton PLD genes. *N—A, C, G or T; K—G or T; M—A or C; R—A or G; W—A or T; Y—C or T
(XLSX)
