# Supplementary figures and images for: Genome-Wide Comparative Analysis of the Phospholipase D Gene Families among Allotetraploid Cotton and Its Diploid Progenitors
Source: PLoS One. 2016 May 23;11(5):e0156281. doi: 10.1371/journal.pone.0156281 (PMC4877076; doi:10.1371/journal.pone.0156281)

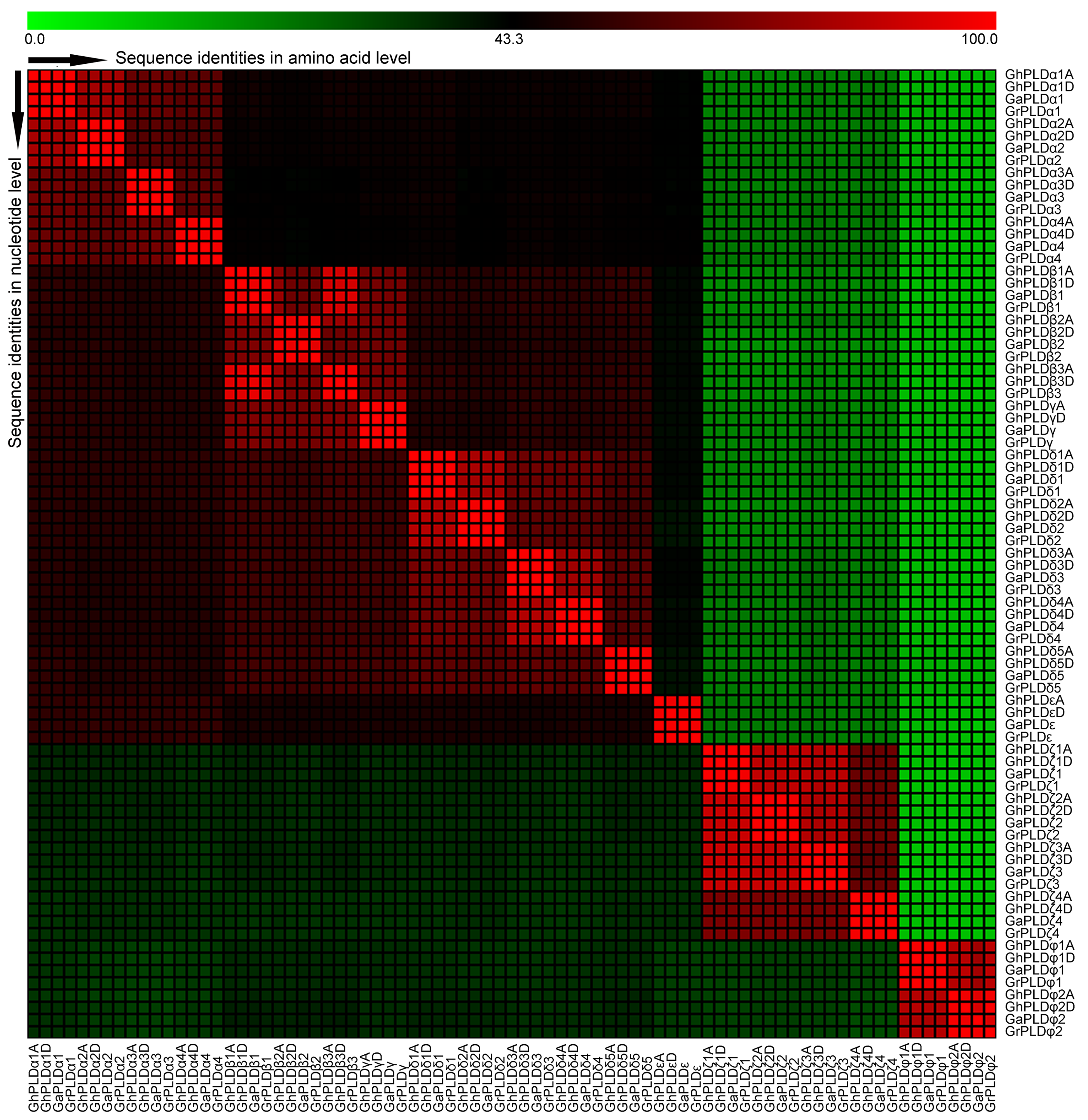

Supplement: S1 Fig — The sequence identities of cotton PLDs at both the nucleotide and amino acid level are calculated with the program DNASTAR. The color scale at the top of the heat map indicates the levels of the sequence identities where light green indicates low and red indicates high. The data at the diagonal lines are equal to 100%. (TIF) [file pone.0156281.s001.tif]

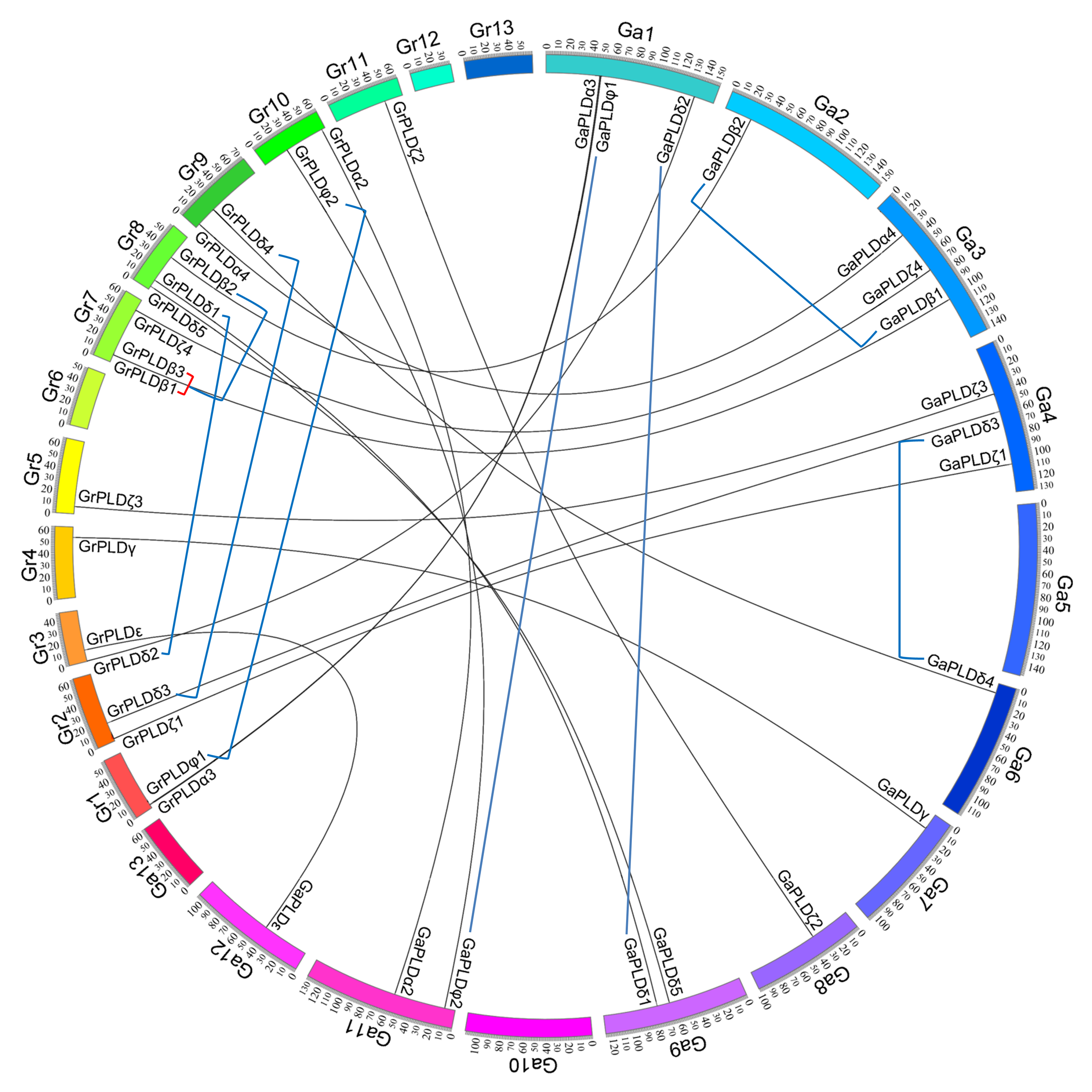

Supplement: S2 Fig — The syntenic relationships between GaPLDs and GrPLDs according to the CottonGen and the Phytozome v9.1 database are illustrated using the program Circos. Tandem and segmental duplicated PLD genes are connected by red and blue lines. The chromosomes of G. arboreum and G. raimondii are designated as Ga1—Ga13 and Gr1—Gr13, respectively. (TIF) [file pone.0156281.s002.tif]

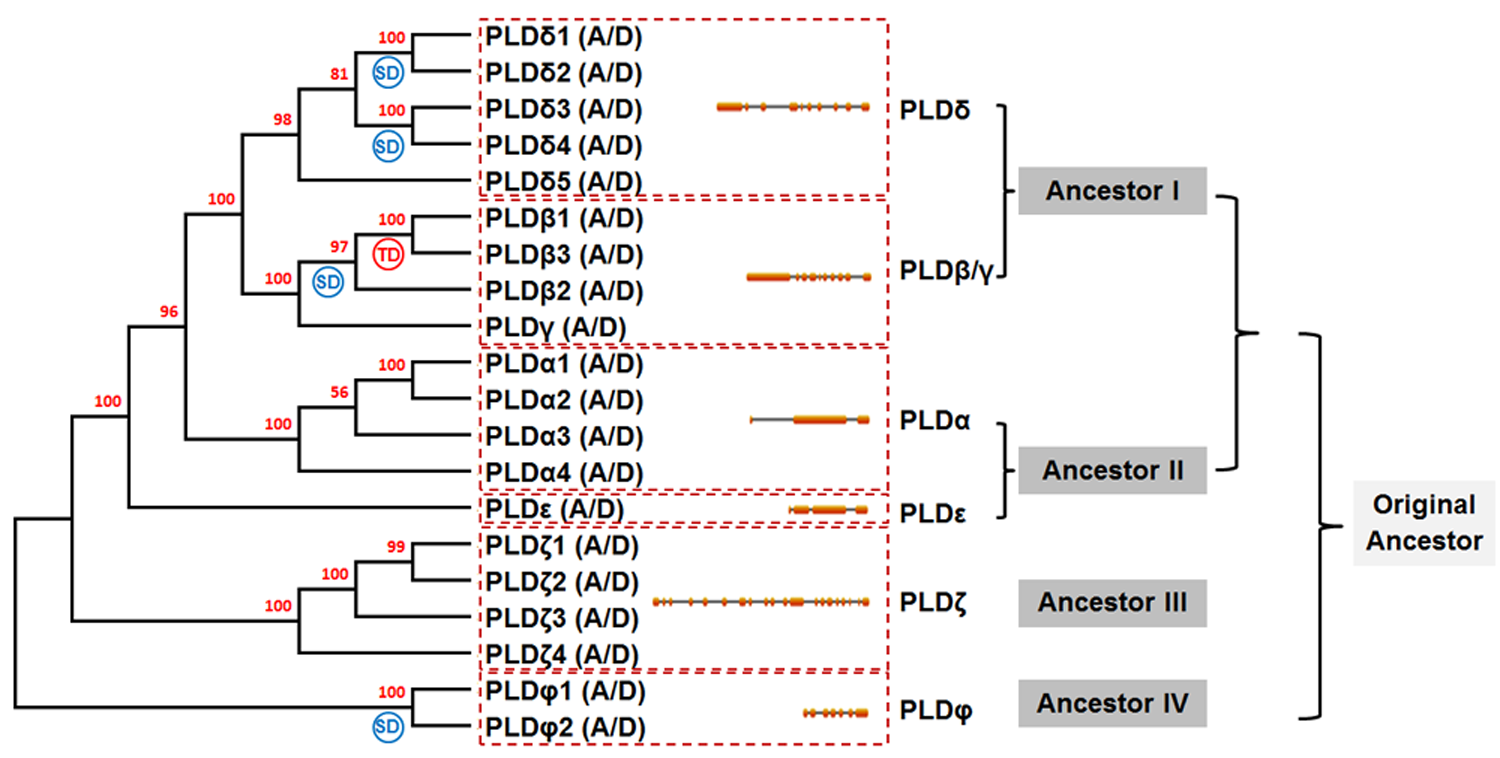

Supplement: S3 Fig — The left column represents the phylogenic relationships of allotetraploid cotton PLDs constructed using the NJ method. Letters in the circles indicate the identified gene duplication event (TD, tandem duplication; SD, segmental duplication). The middle column is the representative exon-intron organization of the PLD subgroup. PLDxA and PLDxD represent G. hirsutum PLDxA and G. hirsutum PLDxD genes, respectively. The right column indicates the putative common ancestors of allotetraploid cotton PLD subgroups. (TIF) [file pone.0156281.s003.tif]
